# An Asian Validation of the TIMI Risk Score for ST-Segment Elevation Myocardial Infarction

**DOI:** 10.1371/journal.pone.0040249

**Published:** 2012-07-16

**Authors:** Sharmini Selvarajah, Alan Yean Yip Fong, Gunavathy Selvaraj, Jamaiyah Haniff, Cuno S. P. M. Uiterwaal, Michiel L. Bots

**Affiliations:** 1 Clinical Epidemiology Unit, Clinical Research Centre, Kuala Lumpur Hospital, Kuala Lumpur, Malaysia; 2 Julius Center for Health Sciences and Primary Care, University Medical Center Utrecht, Utrecht, The Netherlands; 3 Julius Centre University of Malaya, Department of Social and Preventive Medicine, University of Malaya, Kuala Lumpur, Malaysia; 4 Clinical Research Centre, Sarawak General Hospital, Kuching, Sarawak, Malaysia; 5 Department of Cardiology, Sarawak General Hospital Heart Centre, Kuching, Sarawak, Malaysia; 6 National Heart Association of Malaysia, Heart House, Kuala Lumpur, Malaysia; Policlinico San donato milanese, Italy

## Abstract

**Background:**

Risk stratification in ST-elevation myocardial infarction (STEMI) is important, such that the most resource intensive strategy is used to achieve the greatest clinical benefit. This is essential in developing countries with wide variation in health care facilities, scarce resources and increasing burden of cardiovascular diseases. This study sought to validate the Thrombolysis In Myocardial Infarction (TIMI) risk score for STEMI in a multi-ethnic developing country.

**Methods:**

Data from a national, prospective, observational registry of acute coronary syndromes was used. The TIMI risk score was evaluated in 4701 patients who presented with STEMI. Model discrimination and calibration was tested in the overall population and in subgroups of patients that were at higher risk of mortality; i.e., diabetics and those with renal impairment.

**Results:**

Compared to the TIMI population, this study population was younger, had more chronic conditions, more severe index events and received treatment later. The TIMI risk score was strongly associated with 30-day mortality. Discrimination was good for the overall study population (c statistic 0.785) and in the high risk subgroups; diabetics (c statistic 0.764) and renal impairment (c statistic 0.761). Calibration was good for the overall study population and diabetics, with χ2 goodness of fit test p value of 0.936 and 0.983 respectively, but poor for those with renal impairment, χ2 goodness of fit test p value of 0.006.

**Conclusions:**

The TIMI risk score is valid and can be used for risk stratification of STEMI patients for better targeted treatment.

## Introduction

Risk stratification is important in acute coronary syndromes (ACS). It provides information to both patients and clinicians on the possible prognosis and serves as a guide to aggressiveness of treatment [1], [2]. ST-segment elevation myocardial infarction (STEMI) forms the severest spectrum of ACS [3] and the best clinical outcomes are achieved when the primary percutaneous coronary intervention (PCI) strategy is applied [4], [5].

In developing countries, where there is a wide variation of healthcare service provision, it is often challenging to provide the best treatment strategies recommended in international guidelines. In this respect, risk stratification of patients with STEMI takes on greater importance, especially for those at the highest risk strata, such that the most resource intensive strategies can be applied to achieve the greatest clinical benefit.

The Thrombolysis In Myocardial Infarction (TIMI) risk score was developed as a bedside tool to stratify STEMI patients eligible for reperfusion by their mortality risk [6]. This low cost risk estimation may be very suitable for use in developing countries. It was developed in a clinical trial population, and has been validated in non-selected Western patient populations [7], [8]. The TIMI risk score has shown to provide good discrimination in predicting mortality at 30 days and even up to 365 days. This offers some evidence for its clinical applicability in risk stratification and prognostication. However, it is not known how the TIMI risk score performs in a population with many characteristic differences from the population the risk score was derived from, in the era where an early invasive strategy for re-vascularisation is becoming more common. In Malaysia, patients presenting with STEMI are younger, have a much higher prevalence of diabetes, hypertension and renal failure, and present later to medical care than their western counterparts [9].

In this study, we studied whether the TIMI risk score can be applied, i.e., results in adequate risk assessment, in a multi-ethnic Malaysian population presenting with STEMI. We also sought to determine if the TIMI risk score was useful prognostically in subgroups of patients with diseases that are more prevalent in the country and at higher risk of mortality; diabetics [10] and those with renal impairment [11].

## Methods

The National Cardiovascular Disease Database (NCVD) in Malaysia is an on-going observational prospective registry of patients who present with ACS. It commenced on the 1^st^ of January 2006. Patient recruitment occurs at 16 hospitals with varying facilities; 14 from the Ministry of Health, 1 university hospital and the National Heart Institute of Malaysia. All patients aged 18 and above with ACS at these sites have details of their past medical history, presenting symptoms, in-patient clinical care and health outcomes till 1 year post ACS recorded.

### Ethics Statement

The NCVD is registered in the National Medical Research Register of Malaysia (ID: NMRR-07-38-164) and received ethical approval from the Ministry of Health Medical Research and Ethics Committee. A waiver of informed consent was obtained from the Ministry of Health Medical Research and Ethics Committee. Instead, a public notice is displayed at all sites and patients are given the option to opt out of the NCVD.

This study made use of anonymized data from patients who presented with STEMI registered from 1^st^ January 2006 till 31^st^ December 2008 with follow up details recorded till 31^st^ December 2009.

The diagnosis of STEMI is based on the following; signs and symptoms of ACS (chest pain or overwhelming shortness of breath), elevated serum cardiac biomarkers and an ST elevation in contiguous leads of the electrocardiogram or the development of a new left bundle branch block (LBBB) [12]. All clinical care given to patients presenting with STEMI was at the discretion of the treating physician or cardiologist at the respective sites. Diabetes mellitus (DM) status was determined based on self report, or use of blood sugar lowering agents (oral or insulin). Renal impairment was determined based on medically documented reports of moderate to severe chronic kidney disease (CKD); CKD Stage 3 and above (e-GFR below 60 ml/min). For this study, a previous medical history that was noted to be ‘not known’ or ‘not recorded’ was classified as absent.

The TIMI risk score for STEMI was developed using the study population from the Intravenous nPA for Treatment of Infarcting Myocardium Early II (InTIME II) trial [13]. The study population of the InTIME II trial will be referred to as the ‘TIMI development’ population for this study. The elements of the TIMI risk score are age, systolic blood pressure, heart rate, Killip classification, infarct location or left bundle branch block, history of diabetes, hypertension or angina pectoris, weight and time to treatment. The TIMI STEMI scoring mechanism has been published [6]. For this study, the TIMI risk score is slightly modified for ‘time to treatment’ variable. Time to treatment is defined as time from presentation (not symptom onset) to reperfusion, either via thrombolytics (door-to-needle time) or primary percutaneous coronary intervention (door-to-balloon time). Those who did not receive reperfusion therapy for the following reasons; missed thrombolysis (12.6%), thrombolysis was contraindicated (4%) or patient refused treatment (0.2%), were given a score of 1 for time to treatment.

The outcome of interest was 30-day mortality. Details on mortality were obtained via hospital records and a 30-day follow up phone call to the patient/relatives. Confirmation of mortality is done yearly via record linkages with the Malaysian National Registration Department for deaths in the country. The IBM® InfoSphere® QualityStage (http://www-01.ibm.com/software/data/infosphere/qualitystage/) was used for record matching purposes. Rule sets for record matching were prepared based on some of the methods available in the software (such as ‘String character or phrase comparison’, ‘Phonemic name comparison’, ‘Specialised numeric comparisons’, ‘Absolute difference comparison’, etc). The rule sets were implemented for key identifier fields such as name, identification card number, year and month of birth. Accurate record linkages are possible because all Malaysians have a unique numerical identification number. This unique identification number is used for all official matters; including hospital and clinic visit registrations, as well as death registration.

Missing data was checked to determine if it was Missing At Random using the separate variance *t* test. Seven variables with missing values were imputed. Those with missing values of <5% (age 0.1%, systolic blood pressure 1.1%, heart rate at presentation 2%, sex 2.2% and smoking status 4.9%) were imputed using mean or median values where applicable. Two variables with >5% missing (time to treatment, 23.9% and weight, 36.1%) were imputed using single imputation with a random error term method.

A multivariable logistic regression model was used to determine the risk association of the TIMI risk score and 30-day mortality. Odds ratios (OR) and its 95% confidence intervals (95% CI) are reported. All variables included in the original TIMI development set model were included in the validation model [6].

Validity of the TIMI risk score was tested using discrimination and calibration. Discrimination was assessed using the concordance statistic (*c* statistic) which is equivalent to the area under the Receiver Operating Characteristic (ROC) curve. A *c* statistic value of >0.75 is considered good discrimination. Calibration was determined graphically by plotting the observed 30-day mortality rates with the predicted rates which were determined from the observed mortality rates from the TIMI risk score development set. A chi square goodness-of-fit test was used to determine if the observed mortality rates differed significantly from the expected [14]. A p value of <0.05 was considered to be statistically significant. All analyses were performed using SPSS Statistics Version 17.0 for Windows (SPSS Inc., Chicago, Il, USA).

## Results

There were 10682 patients registered in the NCVD registry for ACS from 1^st^ January 2006 till 31^st^ December 2008. Of these, 478 (4.5%) did not have a diagnosis for type of ACS; STEMI, non-STEMI or unstable angina. There were 4701 patients diagnosed with STEMI. Among them, 36.3% had diabetes mellitus and 3.3% had renal impairment. Among those with STEMI, 72.7% were given thrombolysis reperfusion therapy (97.7% streptokinase) and 6.8% had primary PCI. The remainder had either missed thrombolysis (12.6%), or thrombolysis was contraindicated (4%), patient refused thrombolysis (0.2%) or data was missing (3.5%).

Baseline characteristics of the study population and TIMI score derivation population are shown in Table 1. Overall, patients presenting with STEMI in this validation set were younger; only 25% of them being older than 65 years of age, except for those with renal impairment (41%). The population in this study had more chronic conditions such as diabetes, hypertension, prior angina and history of cerebrovascular disease. Presenting characteristics at time of myocardial infarction were also more severe. Higher proportions of the study population had poorer Killip classes, high heart rates (>100 beats per minute) and low systolic blood pressures (<100 mmHg).

**Table 1:** Baseline characteristics of patient populations from TIMI Score development set and study set.

	Overall			TIMI score
Variables	Population	Diabetics	Renal imp	development pop
n	4701	1707	156	15060
**Demographics**				
Age (years)	56 (48, 65)	57 (50, 65)	63 (54, 70)	62 (52, 70)
*>75 y*	7	6.7	10.9	13.7
*65–74 y*	18	18.9	30.1	28.1
Female	15.1	22.4	28.8	24.7
Weight (kg)	67 (58, 77)	68 (58, 79)	62 (53, 76)	77 (69, 86)
*<67 kg*	49.4	47.5	63.5	19.2
Race				
*Malay*	52.9	49.3	44.2	NA
*Chinese*	20.3	16.9	25.6	NA
*Indian*	18.2	26.8	17.3	NA
*Others*	8.6	7	12.8	NA
**Risk factors**				
Smoking status				
Current	50.8	38.9	24.4	44.7
Past	20.1	21.4	26.9	26.4
Never	29.1	39.7	48.7	28.4
Diabetes	36.3	100	62.8	13.9
History of hypertension	48.4	67.8	79.5	30.4
Renal impairment(Mod- severe)	3.3	5.7	NA	NA
Cardiovascular history				
*Prior myocardial infarction*	9.6	12.5	23.1	16
*Peripheral vascular disease*	0.3	0.5	3.2	5.2
*Cerebrovascular disease*	2.7	4	8.3	1
*Prior angina*	51.7	55.1	52.6	21.2
*Documented CAD >50%*	5.7	8.9	16.7	7.2
*Diabetes/HPT/Prior angina*	79.2	100	94.2	47.6
Medications at presentation				
*β-blockers*	12.9	18.3	31.4	15.6
*Calcium channel blockers*	5.1	8.2	21.2	15.7
*Lipid lowering*	16.6	25.1	42.9	9.3
*Anti-arrhythmic*	1.9	2.3	3.2	1.3
**Presenting characteristics**				
Infarct location				
*Anterior or LBBB*	59.1	60.3	57.7	42.7
*Inferior*	45.4	44.4	46.2	56.9
Killip class II–IV	28.9	31.8	44.2	12.6
Heart rate (bpm)	80 (68, 96)	85 (71, 100)	89 (69, 108)	74 (63, 86)
Heart rate >100 bpm	17.8	23.3	30.1	7.7
Systolic blood pressure(mmHg)	133 (115, 152)	134 (115, 156)	134 (113, 160)	140 (122, 155)
Systolic BP <100 mmHg	8.5	8.1	7.7	2.6
Time to treatment >4 hours	35.9	37.6	51.3	24.3

Data are % for categorical variables and median (interquartile range) for continuous variables.

CAD, coronary artery disease, HPT, hypertension, LBB, left bundle branch block.

NA, not available/not applicable.

The diabetics comprised of more Indian patients than the overall study population, had more females and had higher rates of hypertension, prior myocardial infarctions and renal impairment. Compared to others in the study population, patients with renal impairment were of older age, had more severe disease (diabetes, hypertension, prior myocardial infarctions, documented coronary artery disease) and presented with more severe heart failure at the time of myocardial infarction.

The 30-day mortality rate for the study population was 11.1%, of which 9.4% was attributed to in-hospital mortality. The 30-day mortality rate for the TIMI development population was 6.7% [6]. The overall mortality rate among diabetics and those with renal impairment was 14% and 27.6% respectively.

Table 2 presents the risk associations of 30-day mortality for each characteristic used in the TIMI risk score calculation. The risk associations were similar between this validation set and the development set for all characteristics except for age older than 75 years and weight.

**Table 2:** TIMI risk score, characteristics and risk of 30-day mortality.

Characteristics	TIMI risk score *	TIMI Adjusted OR (95% CI) *	Malaysian Adjusted OR (95% CI)
Age ≥75 years	3; 2 (65–74)	2.7 (2.2–3.2)	6.1 (4.5–8.3)
Systolic blood pressure <100 mmHg	3	2.7 (1.9–3.8)	4.3 (3.3–5.6)
Heart rate >100 bpm	2	2.3 (1.9–2.8)	2.7 (2.2–3.4)
Killip class II – IV	2	2.3 (1.9–2.7)	2.8 (2.3–3.5)
Anterior MI or LBB	1	1.6 (1.4–1.9)	1.3 (1–1.6)
Weight <67 kg	1	1.4 (1.2–1.7)	0.8 (0.6–1)
Time to treatment >4 hours	1	1.4 (1.2–1.6)	1.3 (1.1–1.7)
**Diabetes		1.4 (1.2–1.7)	1.4 (1.1–1.7)
**History of HPT	1	1.3 (1.1–1.5)	1.2 (0.9–1.5)
**Prior angina		1.4 (1.1–1.6)	0.9 (0.7–1.1)

MI indicates myocardial infarction, LBB, left bundle branch block, HPT, hypertension.

Other variables adjusted in the model: never smoked, prior MI, peripheral arterial disease, anti-arrhythmic medication, lipid lowering drugs and female sex.

* obtained from Morrow et al (6).

** Diabetes, HPT and prior angina combined has a risk score of 1.

**Figure 1:**
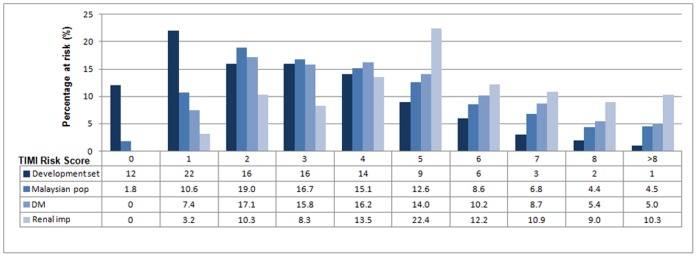
Percentage at risk by the TIMI risk score for the TIMI risk score development population, Malaysian STEMI population, as well as diabetic (DM) and renal impairment sub-groups.

Figure 1 depicts the distribution of the TIMI risk score for the TIMI development population and the Malaysian validation population. It clearly shows a difference in the risk distribution, with the Malaysian population having higher proportions of intermediate risk (TIMI risk score 4–6) and high risk categories (TIMI risk score of ≥7), including the diabetic and renal impairment subgroups.

**Figure 2:**
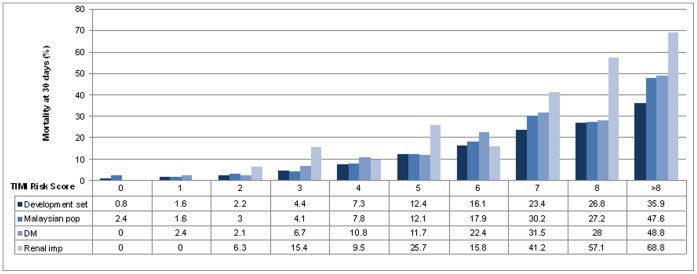
Mortality rate at 30 days for the TIMI risk score development, Malaysian STEMI population, as well as diabetic (DM) and renal impairment sub-groups.

There was a strong association of increasing risk of 30-day mortality with each increasing TIMI risk score for the study population, *p* value of <0.001 (Figure 2). This was consistent for the diabetic and renal impairment subgroups (*p* values <0.001 for tests of trend). The mortality rate for each risk score ranged from 2.4 to 100% from the lowest TIMI risk score (0) to the highest (13). For diabetics, the mortality rate ranged from 2.4 to 72.7% from the lowest TIMI risk score (0) to the highest (11) and for those with renal impairment, it ranged from 0 to 100% (risk score 0 to 11).

Discrimination for the TIMI risk score for this study population was good, *c* statistic 0.785 (95% confidence limit 0.77, 0.81) and also performed better than the original development set (*c* statistic 0.779) [6]. This good discrimination was consistent in both the diabetic and renal impairment subgroups; c statistic 0.764 (0.73, 0.80) and 0.761 (0.68, 0.85) respectively. Calibration of the TIMI risk score was good for the overall study population and diabetics (Figure 2) with a χ2 goodness-of-fit test p value of 0.936 and 0.983 respectively. However, there was poor calibration for the renal impairment subgroup; χ2 goodness-of-fit test p value of 0.006.

## Discussion

Our study provides a fully independent external validation of the TIMI risk score, which includes geographic and temporal validation. It confirms that the TIMI risk score can be used to accurately risk stratify patients presenting with ST-elevation myocardial infarction in Malaysia, despite having more severe presenting characteristics than the original TIMI risk score population. Aside from that, risk stratification worked well for high risk groups prevalent in Malaysia; the diabetics and those with renal impairment.

Although the management options for patients with STEMI are well established [4], [15], [16], our findings are important. Firstly, clinicians managing life-threatening STEMI conditions can accurately risk stratify patients and discriminate those who are more likely to benefit from primary PCI or thrombolytics, depending on the resources available at hand. A recent pooled meta-analysis confirmed that absolute risk reduction for mortality using thrombolytics or primary PCI depended on the patient’s baseline risk [17]. Other studies have shown that in low risk patients, fibrinolysis [18] and even conservative therapy [19] performed as well as primary PCI for 30-day mortality. Therefore, the use of the TIMI risk score in Malaysia may help improve the use of limited resources through better targeted treatment for higher risk patients.

Secondly, it is beneficial to clinicians in the Malaysian setting to be able to use an existing risk score for stratification instead of developing a new tool. There have been various studies developing new scores or identifying additional biomarkers which utilize resource intensive or time-consuming investigations such as C-Reactive Protein [20], B-type natriuretic peptide [21], creatinine (GRACE risk score) [22], [23] and platelet function [24]. The validation of this risk score which uses signs, symptoms and investigations readily done at the time of presentation provides an efficient inexpensive tool for risk stratification. This is essential in developing countries where publicly funded health care systems cater to the majority of the population [25].

Finally, our findings are also relevant to government policy makers and fund holders who are involved in providing care for patients with STEMI. For Malaysia, there is a relative lack of hospitals with cardiac care facilities adequately equipped and resourced for primary PCIs, compared to developed countries [26], [27]. At present, heavily subsided public-access cardiac care facilities exist primarily in large urban areas. The validation of the TIMI risk stratification score in the Malaysian population can provide policy makers and fund holders with information on the distribution of high-risk STEMI patients. Regions with higher numbers/proportions of high-risk patients may benefit from the addition of primary PCI resources at existing facilities. We believe risk stratification can be used to support the process of healthcare planning in Malaysia, from the perspective of STEMI management.

The TIMI risk score for STEMI patients has been validated in Western populations [7], [8]. It has even been compared to different risk scores for STEMI such as the CADILLAC, GRACE and PAMI score and has been shown to have high prognostic accuracy [28], [29]. Our findings confirm the validity of the TIMI risk score in STEMI for an Asian multi-ethnic population.

This study has various strengths. Firstly, it is a prospective multi-centre study with its population comprising of various ethnic groups such as the Malays, Chinese and Indians, which comprise of ethnicities of a large part of Asia. Secondly, this study population comprised a broad spectrum of STEMI patients unlike those found in clinical trials. Thirdly, mortality events were reconfirmed by linkages with the National Registration Department for deaths. Therefore, even for patients lost to follow-up, information on mortality was still recorded in the database.

The TIMI risk score development population consisted of STEMI patients undergoing a clinical trial comparing lanetoplase versus alteplase. It is worth noting that in our study population, the majority of patients (73%) had reperfusion therapy by fibrinolytics (mainly streptokinase) with a relatively low rate of primary PCIs (<7%) as the first line of management for STEMI. Our findings suggest that the TIMI risk score covers the more important variables that affect short term prognosis, irrespective of the type of treatment given. Our management of STEMI is consistent with that in other developing countries [30]. While major improvements to our healthcare system is planned, such as improving human resource (experienced, trained cardiologists), physical and financial infrastructure [31], reperfusion using the fibrinolytic strategy remains the mainstay of STEMI management in Malaysia. Therefore our findings are relevant to our country and may be to others at a similar evolutionary stage of cardiovascular healthcare provision.

Our study had a high percentage of missing values for two variables used in the TIMI risk score; time to treatment and weight. Studies have shown that complete case analysis leads to biased estimates of risk relations [32], [33], hence imputation was used. Both these variables have a low score of one and are independent of each other. Therefore, we anticipate any misclassification of one of the variables to be a non-differential misclassification bias. In other words, the TIMI risk score can still be used for risk stratification (relative ranking), but it may not accurately predict mortality rates by the different risk scores.

The poor calibration seen in the renal impairment subgroup is probably due to the small sample size of 156 patients and thus limits its interpretation. Further validation in this subgroup is warranted. However, this poor calibration seen is consistent with published findings of the TIMI risk score validation in non-STEMI patients [34].

The higher overall mortality rate observed in our validation population compared to the TIMI development population is due to the larger proportion of high risk patients and their higher mortality rates. This higher mortality rate seen among the high-risk strata needs to be investigated further.

In conclusion, the TIMI risk score is relevant and may be of benefit in improving clinical care through better targeted treatment, for patients presenting with ST-segment elevation myocardial infarction in Malaysia.
